# Effect of Ultrasonic Induction on the Main Physiological and Biochemical Indicators and *γ*–Aminobutyric Acid Content of Maize during Germination

**DOI:** 10.3390/foods11091358

**Published:** 2022-05-07

**Authors:** Liangchen Zhang, Nan Hao, Wenjuan Li, Baiqing Zhang, Taiyuan Shi, Mengxi Xie, Miao Yu

**Affiliations:** 1 Institute of Food and Processing, Liaoning Academy of Agricultural Sciences, Shenyang 110161, China; napoleon19831214@163.com (L.Z.); sty1965fightting@163.com (T.S.); moor1112@163.com (M.X.); 2 Corn Research Institute, Liaoning Academy of Agricultural Sciences, Shenyang 110161, China; lnhn1981@163.com; 3 College of Food Science and Technology, Shenyang Normal University, Shenyang 110034, China; lwj20211111@163.com (W.L.); zbqfood@126.com (B.Z.)

**Keywords:** ultrasonic, maize, germination, physiological and biochemical indicators, *γ*–aminobutyric acid

## Abstract

Research on the nutrient content of cereal grains during germination is becoming a hot topic; however, studies on germinated maize are still scarce. This study aimed to provide a technical reference and theoretical basis for the development of functional maize health foods and to expand the application of ultrasonic technology in the production of germinated grains. In this study, the germination rate of maize was used as the evaluation index, and the ultrasonic frequency, ultrasonic temperature, and induction time were selected as the influencing factors in orthogonal experiments to determine the optimal process parameters for ultrasonic induction of maize germination (ultrasonic frequency of 45 kHz, ultrasonic temperature of 30 °C, and ultrasonic induction time of 30 min). Based on this process, the effects of ultrasonic induction on the main physiological, biochemical, and *γ*–aminobutyric acid contents of maize during germination were investigated. The results showed that the respiration of the ultrasonic treated maize was significantly enhanced during germination, resulting in a 27% increase in sprout length, as well as a 4.03% higher dry matter consumption rate, and a 2.11% higher starch consumption rate. Furthermore, the reducing sugar content of germinated maize increased by 22.83%, soluble protein content increased by 22.52%, and *γ*–aminobutyric acid content increased by 30.55% after ultrasonic induction treatment. Throughout the germination process, the glutamate acid decarboxylase activity of the ultrasonically treated maize was higher than that of the control group, indicating that ultrasonication can promote maize germination, accelerate the germination process, and shorten the enrichment time of *γ*–aminobutyric acid in germinated maize. The results of this study can be applied to the production of *γ*–aminobutyric acid enrichment in germinated maize.

## 1. Introduction

Maize (*Zea mays* L.) is an annual cereal crop which produces kernels that are rich in nutrients, such as unsaturated fatty acids, glutathione, dietary fiber, vitamins, trace elements, protein, fat, and carotene [1]. However, maize protein has poor nutritional availability, with a utilization rate of only 6% in the food industry [2]. Modifying germination conditions can enhance their nutritional quality, reduce levels of antinutritional factors, and improve their taste [3]. Previous studies have confirmed that the *γ*–aminobutyric acid (GABA) content of brown rice, soybean, and fava bean increased significantly after germination [4]. GABA is an inhibitory neurotransmitter in mammalian cerebrospinal fluid that has a variety of health benefits [5]. GABA has been shown to inhibit cancer cell proliferation and stimulate apoptosis, regulate blood pressure and blood cholesterol, and reduce pain and anxiety [6]. Plant derived GABA products have received much attention for their safety and natural properties.

GABA is mainly from glutamate catalyzed by glutamate decarboxylase (GAD), and the activity of GAD is the main factor affecting the content of GABA in plants [7]. GAD is an endogenous enzyme in plant cells. Under environmental stimuli, such as hypoxia, darkness, ultraviolet light, salt stress, and mechanical vibration, its activity results in an increase in GABA content [4].

Ultrasonic waves refer to elastic waves propagated in gas, liquid, solid, and other media by mechanical vibration at a certain frequency. Ultrasonic waves can generate not only mechanical force but also thermal effects that cause various physical and chemical changes, and ultrasonic waves of appropriate intensity acting on biological tissues can affect plant metabolism and biochemical properties [8]. Ultrasonic waves have sterilizing effects which can eliminate bacteria on the surface of seeds, reduce bacterial infection of seeds, break the dormant period of seeds, and improve the vitality and germination rate of seeds [9]. Ultrasonic induction can accelerate the division and growth of plant cells and the protein synthesis of protoplasts by destroying the structure of biological macromolecules, increasing the pH value and Ca^2+^ concentration of cells, and changing the permeability of cell membranes to shorten the germination time of plant seeds [10]. Ultrasonic induction can stimulate the activity of endogenous enzymes in seeds; however, the activities of endogenous enzymes in seeds were found not to increase immediately after induction by ultrasonic waves but to increase significantly during germination compared with an untreated group [11]. Activation of endogenous enzymes is beneficial for the enrichment of functional factors in seeds [12]; studies have shown that when rice, soybean, mungbean, and other plant food raw materials are induced by ultrasonic waves and then germinated, the GABA content increases significantly [4]; the total phenolic content increased significantly in barley germinated after ultrasonic induction [8], while the content of resveratrol was significantly increased in peanuts germinated after ultrasonic induction [11]. The activation of endogenous enzymes induced by ultrasound can reduce or eliminate the concentration of some antinutrients, such as phytic acid and protein inhibitors, contained in grains and beans, increase their protein digestibility and bioavailability, and significantly improve nutritional quality [13,14].

Ultrasonic induction technology can improve the yield of germinated maize and improve its nutritional quality. However, the research on the artificial regulation of maize germination under stress has not been reported. In this work, we investigated the effect of ultrasonic induction treatment on maize germination rate, physiological and biochemical indicators, and GABA content. Additionally, the relationships between glutamic acid (Glu) content, Glutamate Decarboxylase (GAD) activity, and GABA content were investigated. This study aimed to provide a technical reference and theoretical basis for the development of functional maize health foods and to expand the application of ultrasonic technology in the production of germinated grains.

## 2. Materials and Methods

### 2.1. Materials and Reagents

For this work, the maize variety Liaodan 575 was provided by the Maize Research Institute of the Liaoning Academy of Agricultural Sciences. The maize seeds were harvested in October 2020, encapsulated in airtight containers, and stored in a sealed container at 4 °C. All chemicals and solvents used in this study were purchased from Sigma–Aldrich (St. Louis, MO, USA) or Solarbio (Beijing Solarbio Science & Technology Co., Ltd., Beijing, China).

### 2.2. Preparation for Maize Germination

#### 2.2.1. Maize Soaking Treatment

The maize soaking treatment was assessed as described by Yu et al. [11], with minor modifications. A total of 300 g of maize seeds were weighed and sterilized by soaking in 1% by volume NaClO solution (Beijing Solarbio Science & Technology Co., Ltd., Beijing, China) for 30 min, rinsed with deionized water to restore neutral pH, and then soaked in an incubator for 6 h at 30 °C. Another 300 g of maize seeds were treated in the same way and then used as the control.

#### 2.2.2. Ultrasonic Induction Treatment of Maize

The soaked maize samples were wrapped in a layer of sterile gauze and placed in an ultrasonic cleaner (KQ–300VDE, overall dimensions: 410 × 350 × 420 mm, tank dimensions: 300 × 240 × 180 mm, ultrasonic power: 250 W, heating power: 600 W, range of temperature: 20–80 ℃ ± 3℃, Kunshan Ultrasonic Instrument Co., Ltd., Kunshan, China) for ultrasonic induction treatment. We utilized three different ultrasonic frequencies: 28 kHz, 45 kHz, and 100 kHz. Additionally, induction times of 10, 20, and 30 min were tested, and temperatures of 30 °C, 35 °C, and 40 °C were utilized. L_9_3^3^ orthogonal testing was carried out to identify the optimum ultrasonic induction treatment. The experimental design is shown in Table 1.

#### 2.2.3. Maize Germination Treatment

A single layer of soaked maize seeds was placed in Petri dishes and kept at a constant temperature of 30 °C and 90% relative humidity for 96 h. Portions of the maize seeds were removed at 0 h, 12 h, 24 h, 36 h, 48 h, 60 h, 72 h, 84 h, and 96 h during germination, rinsed three times with distilled water, and freeze-dried to a moisture fraction of less than 10%. The maize seeds were then stored at 4 °C before testing.

### 2.3. Methods

#### 2.3.1. Determination of Germination Rate and Sprout Length

The germination rate was calculated as described previously [15]. Twenty-five maize samples were randomly selected at different time points and sprout lengths were measured using a vernier caliper (500–196–30, Mitutoyo, Tokyo, Japan).

#### 2.3.2. Determination of Respiration Intensity

The respiratory intensity was measured as described by He et al. [16], with minor modifications. A total of 25 maize seeds were randomly selected at different time points, placed in a 40 mm × 80 mm jar, and measured with an infrared gas analyser (GXH–3051, Jun Fang Li Hua Technology Research Institute, Beijing, China) at 20 °C. Deoxygenated air was utilized as the carrier gas and measurements were taken for one hour. The respiration intensity was expressed as the mass of CO_2_ released from 1 g of maize respired for 1 h in mg/g·h.

#### 2.3.3. Determination of Dry Matter Content

Seven maize seeds were selected and weighed to determine their fresh weight (M), then dried at 105 °C for 15 min, followed by baking at 80 °C to a constant weight (m). The dry matter content of the maize was then taken as m/M.

#### 2.3.4. Determination of Starch and Reducing Sugar Content

The starch content of maize was determined using the polarimetry method [17] and the reducing sugar content was determined using the 3,5–dinitrosalicylic acid method [18].

#### 2.3.5. Determination of Soluble Protein

Soluble protein content was determined by the Bradford method [19].

#### 2.3.6. Determination of γ–Aminobutyric Acid and Free Amino Acid Content

The determination method for GABA and free amino acid contents was assessed according to the method described by Chen et al. [20] and Shen et al. [21], with some modifications. A 1.0 g sample of germinated maize was crushed and placed in a glass tube containing 5 mL of deionized water. This was then put into an ultrasonic apparatus (PRESET–SW–12H, Sonoswiss, Switzerland) at 40 °C and 45 kHz for 30 min. The sonicated sample was centrifuged in a centrifuge at 8000× *g* for 5 min and the supernatant was collected. The precipitate was then extracted again as described above. The supernatants of the two extractions were combined and a 3 mL sample of the combined supernatants was mixed with 7 mL of 100% ethanol, stored at 4 °C overnight, and centrifuged at 8000× *g* for 10 min. The supernatant was collected and dried under nitrogen (DN–12A, Bilang, Shanghai, China), the dried material was dissolved with 1 mL of 0.02 M HCl (Beijing Solarbio Science & Technology Co., Ltd., Beijing, China), and centrifuged at 8000× *g* for 10 min. The supernatant contained GABA and free amino acid extracts, which were then were filtered through a 0.45 μm syringe filter (0.45 μm, Shanghai Jinlan Instrument Manufacturing Co. Ltd., Shanghai, China), and an automatic amino acid analyzer (L–8900, Hitachi, Tokyo, Japan) was used to measure GABA and free amino acid content.

#### 2.3.7. Determination of Glutamate Decarboxylase Activity

The determination method of GAD activity was assessed according to the method described by Khwanchai et al. [22], with some modifications. A 0.7 g sample of crushed maize was mixed with 3.2 mL of potassium phosphate buffer (0.05 M, pH 5.8). The potassium phosphate buffer contained 2 mM ethylenediaminetetraacetic acid (Beijing Solarbio Science & Technology Co., Ltd., Beijing, China), 2 mM β–mercaptoethanol (Beijing Solarbio Science & Technology Co., Ltd., Beijing, China), and 0.2 mM pyridoxal phosphate (Beijing Solarbio Science & Technology Co., Ltd., Beijing, China). The mixture was centrifuged at 1000× *g* for 20 min at 4 °C, and the resulting supernatant was used as the GAD extract. A 200 μL sample of GAD extract was mixed with 100 μL of 1% Glu (Sigma–Aldrich, St. Louis, MO, USA) and incubated for 2 h at 40 °C, pH 5.8, then the enzyme was inactivated at 90 °C for 10 min in a water bath and the GABA content was measured. The determination method of GABA content was described in Section 2.3.6. One unit of GAD activity was defined as the production of 1 μmol GABA from Glu per min at 40 °C.

#### 2.3.8. Statistical Analyses

All the experiments were performed in triplicate and the results are reported as the means ± standard deviation. Values were analyzed by one-way ANOVA, followed by Duncan’s multiple range tests using Statistical Product Service Solutions 17.0 (SPSS Inc., Chicago, IL, USA), and a difference was considered significant when the *p*-value was < 0.05.

## 3. Results and Discussion

### 3.1. Selection of the Ultrasonic Induction Treatment Process for Germinating Maize

The germination rates of maize treated by different ultrasonic processes are shown in Table 2. The results for the ultrasound group showed that the highest germination rate was achieved at an ultrasonic frequency of 45 kHz and a temperature of 30 °C for a duration of 30 min, with a germination rate of 90.00 ± 1.63%, while the germination rate of maize without ultrasonic induction treatment was 79.50 ± 2.00%. The germination rate of maize after ultrasonic induction treatment was increased by 10.5%. As germination rate is the key to germinating maize preparation, these process parameters were chosen for the ultrasonic induction treatment process for germinating maize and provided a good basis for subsequent experiments. Similar results have been found in other crops, including *Oryza sativa* L. [23], *Capparis spinosa* L. [24], *Giycine max* L. [25], *Triticum aestivum* L. [26], and *Cicer arietinum* L. [27].

The influence of ultrasonic waves on maize germination rate is mainly due to the mechanical vibration and thermal effect of the cavitation effect. The mechanical vibration generated by an ultrasonic wave can accelerate the rate at which water enters maize seed and help the seed to end its dormant state. The thermal effect generated by ultrasonication will accelerate the decomposition and dissolution of maize seeds and provide sufficient nutrients for seed germination. The efficiency of this thermal effect is several times higher than that of ordinary heating. Ultrasound is also a form of energy, which converts electrical energy into kinetic energy in the form of ultrasonic vibration and transmits it to the cells of the seed, stimulates the enhancement of life movement, and accelerates the physiological and biochemical reactions inside the seed [28]. Ultrasonic induction improves the germination rate of maize seeds through biological, chemical and physical methods.

### 3.2. Effects of Ultrasonic Induction Treatment on Physiological and Biochemical Indicators during Maize Germination

#### 3.2.1. Effect of Ultrasonic Induction Treatment on Sprout Length during Maize Germination

**Figure 1:**
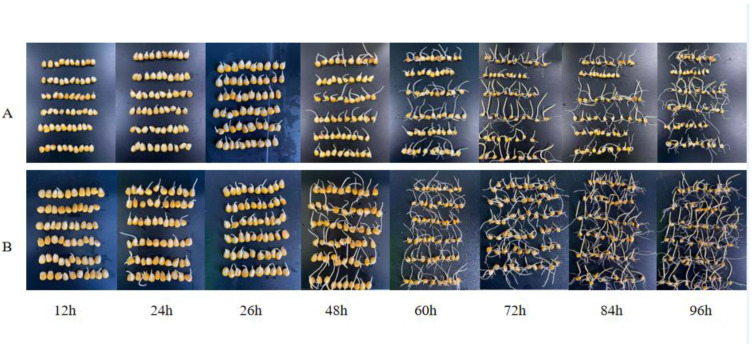
Different stages of germinated maize. (**A**) Control group. (**B**) Ultrasound group.

As shown in Figure 1, the growth pattern of maize in the ultrasound group was significantly better than that of the control group. The fibrous roots of maize in the ultrasound group had already grown at 60 h after germination, while the fibrous roots of maize in the control group only appeared visibly at 96 h after germination, which visually proves that ultrasound induction can promote maize germination.

**Figure 2:**
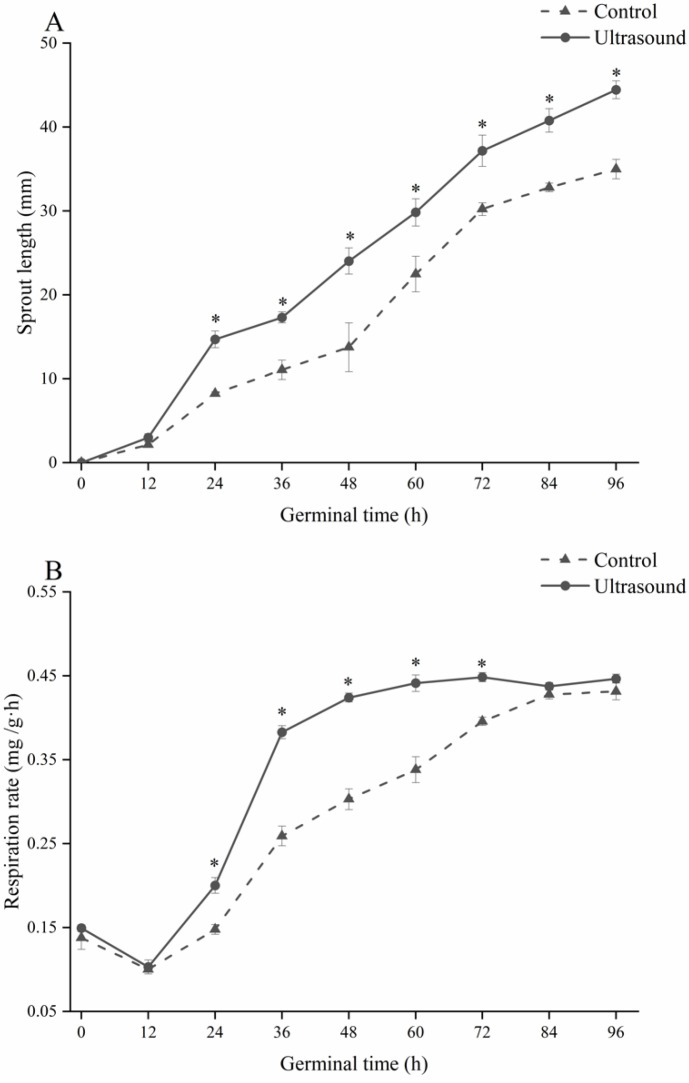
The effect of ultrasound induction treatment on sprout length (**A**) and respiration rate (**B**) during maize germination. * Indicates a significant difference (*p* < 0.05) between the ultrasound and control groups.

As shown in Figure 2A, the sprout length of maize in the ultrasound group was significantly higher than that in the control group from 24 h to 96 h after germination (*p* < 0.05). The sprout length of maize in the ultrasound group was 44.45 ± 1.06 mm at the end of germination, which was 1.27 times longer than that in the control group. Ding et al. [28] found that treating long-grain de-hulled rice (*Oryza sativa* L.) at a 25 kHz ultrasonic frequency for 5 min also significantly increased sprout length.

Ultrasonic induction treatment has also been demonstrated to stimulate cell growth, accelerate protein synthesis in protoplasts, accelerate the induction of plant cell division, promote the growth of plant seed scions, and shorten the germination period of seeds [29,30]. Cavitation generated by ultrasonic induction treatment shatters seed coats and thereby markedly reduces resistance to water diffusion. This not only promotes the passage of water molecules through cell walls and increases the transfer rate of nutrients but also enables the embryo to freely absorb excess water, thereby promoting embryo development and growth [31].

#### 3.2.2. Effect of Ultrasonic Induction Treatment on the Respiratory Rate of Germinating Maize

As shown in Figure 2B, the respiratory intensity of maize in both groups decreased initially, followed by a gradual increase as germination progressed. However, the respiration rate of the ultrasound group increased more strongly compared to the control and was positively correlated with sprout length (r = 0.969, *p* < 0.05).

Enhanced respiration of maize seeds is one of the most significant physiological changes in the germination process; the enhancement of respiration in the ultrasound group also proved that ultrasonic induction can promote the growth and metabolism of maize seeds. The respiratory intensity of maize in both groups in this experiment started to increase after 12 h of germination, which is consistent with trends reported in rice, wheat, and other cereal crops [26,32]. The respiratory intensity of maize was weakened and then increased during the 0–24 h stage, which is related to the predominance of anaerobic respiration in the early germination of maize [33].

#### 3.2.3. Effect of Ultrasonic Induction Treatment on Dry Matter Content of Maize during Germination

**Figure 3:**
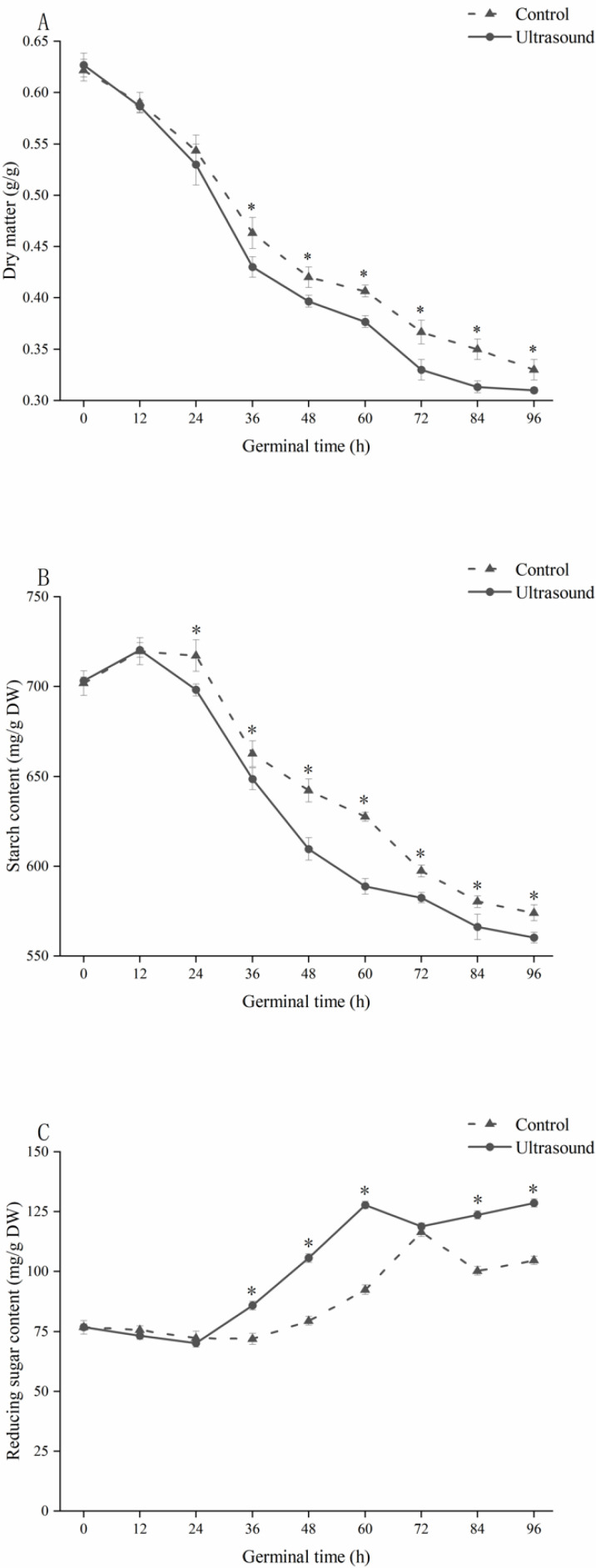
The effect of ultrasound induction treatment on dry matter (**A**), starch content (**B**), and reducing sugar content (**C**) during maize germination. * Indicates a significant difference (*p* < 0.05) between the ultrasound and control groups.

As shown in Figure 3A, the dry matter content in both the control and ultrasound groups showed a similar trend over the course of 96 h. At 96 h, the dry matter content of the ultrasound group was 49.20% of the dry matter content before germination (0.31 ± 0.003 g/g), while that of the control group was 53.23% of the dry matter content before germination (0.33 ± 0.01 g/g).

The dry matter content in the ultrasound group was significantly negatively correlated with respiration intensity (R = −0.987, *p* < 0.05). The increase in respiration during maize germination was accompanied by the rapid consumption of organic matter [34], which explains the increased rate of dry matter reduction seen in the ultrasound group compared to the control group.

#### 3.2.4. Effects of Ultrasonic Induction Treatment on the Starch and Reducing Sugar Contents of Germinating Maize

As shown in Figure 3B, changes in the starch contents of both groups were similar as germination progressed, with a slow initial increase followed by a continuous decrease. The starch content of the ultrasound group was significantly lower than that of the control group from 24 h to 96 h of germination. At 96 h of germination, the starch content of the control group was 574.04 ± 4.41 mg/g, with a starch consumption rate of 18.21%, while the starch content of the ultrasound group was 560.21 ± 2.97 mg/g, with a starch consumption rate of 20.32%. The increase in starch content in the early stages of germination in both groups was correlated with the decrease in dry matter content due to water absorption by the maize, and the continuous decrease in starch content in the middle and late stages of germination was likely due to the activation of endogenous enzymes and hydrolysis of starch [3]. Hu et al. [35] found that maize showed a 4.74 fold increase in α–amylase activity, a 16.82 fold increase in β–amylase activity, and a 14.41 fold increase in total amylase activity during germination.

The starch consumption rate of maize in the ultrasound group was 2.12% higher than that in the control group at the end of germination (*p* ˂ 0.05), which may be related to the ability of ultrasonic induction treatment to increase the endogenous enzyme activity of plant cells and promote cell growth and biosynthesis [36]. Ultrasound has multiple effects on the hydrolysis of starch during the germination of cereal seeds, including altering the active site and configuration of enzymes present in the grain, affecting synthesis and the conversion of enzymes during germination, and changing the microstructure of starch, leading to changes in enzyme sensitivity [37].

As shown in Figure 3C, both control and ultrasound groups showed similar trends in reducing sugar throughout the germination process. Within 0–12 h of maize germination, the content of reducing sugar did not change significantly; after 12 h of germination, the soluble sugar content started to decrease, likely due to growth of the radicle [38], the activation of endogenous enzymes, and consumption of small sugar molecules [39,40]. The fluctuation in soluble sugar content during the germination process in both groups indicated that the synthesis and metabolism of soluble sugars took place simultaneously. The reducing sugar content of maize in the ultrasound group was significantly higher than that in the control group from 36 to 60 h and from 84 to 96 h of germination, with the peak occurring at 60 h of germination, 12 h earlier than that in the control group. The reducing sugar content in the ultrasound group at the end of germination was 128.5 ± 1.68—an increase of 22.83% compared to the control group. This indicates that ultrasonic induction treatment was able to promote carbohydrate metabolism during maize germination, and this promotion effect primarily manifested in the middle and late stages of maize germination.

Reducing sugar in maize mainly comes from the hydrolysis of starch. Starch is hydrolyzed into maltose under the action of amylase, and maltose is hydrolyzed into glucose by maltase. Under the action of germination and ultrasonic induction, the enzyme activity involved in starch hydrolysis is greatly enhanced and is increased by the reducing sugar content in maize during germination. Xia et al. [23] found that brown rice treated with ultrasound showed a more pronounced decrease in starch content and an increase in reducing sugars compared to untreated samples. In addition to enhancing the activity of amylases during seed germination, ultrasound can increase the concentration of substrates for starch hydrolysis reactions by disrupting starch–protein and starch–lipid complexes, thereby accelerating the rate of starch metabolism [41]. These factors also explain why the reducing sugar content of the maize in the ultrasound group was higher than that in the control group during the germination of the maize.

#### 3.2.5. Effect of Ultrasonic Induction Treatment on the Soluble Protein Content of Germinating Maize

**Figure 4:**
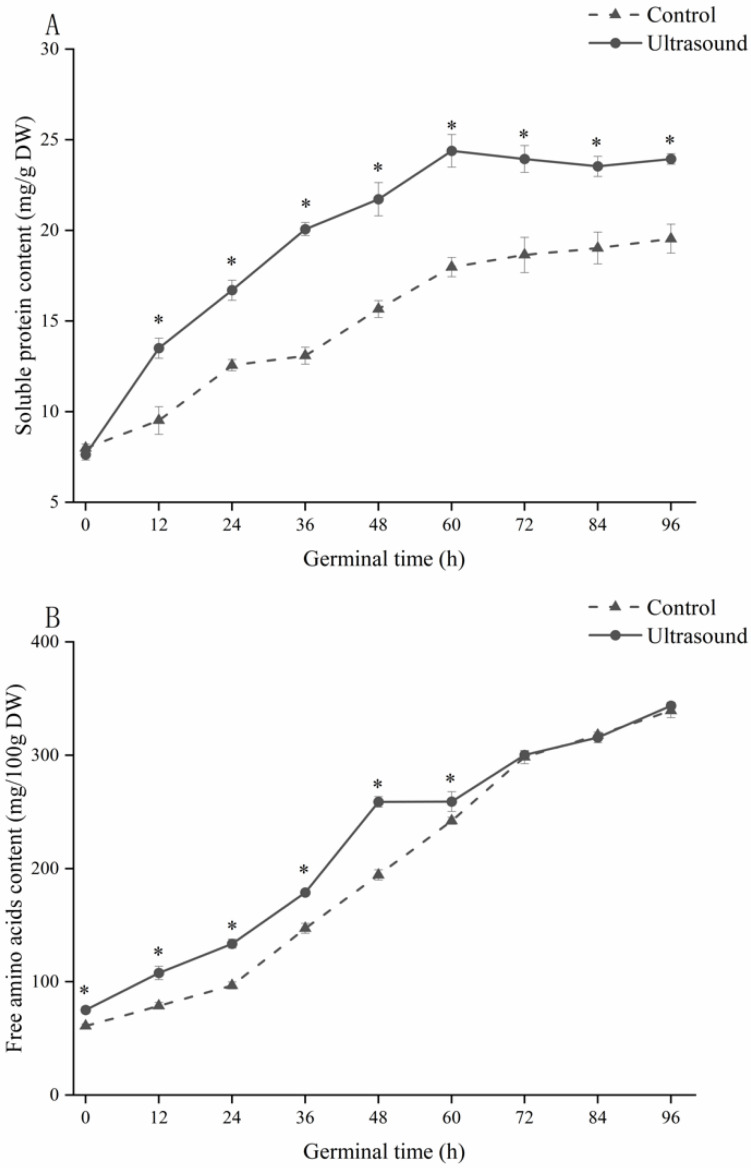
The effect of ultrasound induction treatment on soluble protein content (**A**) and free amino acid content (**B**) during maize germination. * Indicates a significant difference (*p* < 0.05) between the ultrasound and control groups.

As shown in Figure 4A, the peak soluble protein content of maize in the ultrasound group appeared at 60 h of germination, which was 36 h earlier than that of the control group. During germination, the soluble protein content of maize in the ultrasound group was significantly higher than that in the control group. At the end of germination, the soluble protein content of maize in the ultrasound group was 23.94 ± 0.29 mg/100 g, which was 22.52% higher than that in the control group.

The trend of soluble protein content change during the germination of maize in the two groups was different. In the early stage of maize germination, the soluble protein content increased and its source was the degradation of stored proteins; with the conversion of soluble forms and the synthesis of new proteins, the increase in soluble protein content during germination is presumably due to the formation and completion of the enzyme system, which can be regarded as the result of the dissolution of stored proteins under the action of proteases and the synthesis of new protein substances [42]. The rapid increase in the soluble protein content of maize in the ultrasound group during the early stages of germination suggests that the ultrasonic induction treatment accelerated the completion of this process and that the soluble protein content of maize in the ultrasound group stabilized after reaching its peak, probably because the ultrasonic induction treatment brought forward the morphological establishment of the seedlings and balanced protein synthesis and consumption [43]. The reason for this is that, under the action of ultrasound, a variety of physical mechanical effects are generated between the cell wall of plant cells and the liquid medium and these mechanical effects change the physical structure and integrity of plant cells [33,44].

#### 3.2.6. Effect of Ultrasonic Induction Treatment on the Free Amino Acid Content of Germinating Maize

As shown in Figure 4B, the free amino acid content of maize in both the control and ultrasound groups continued to increase during germination, and the free amino acid content of maize in the ultrasound group was significantly higher than that in the control group from 0 to 60 h of maize germination (*p* < 0.05). The free amino acid content of maize in the ultrasound group increased from 75.13 ± 2.74 mg/100 g to 343.71 ± 2.94 mg/100 g, indicating that ultrasound promotes protein hydrolysis in maize. Similar findings were obtained by Ding et al. [45], who applied ultrasound to germinating red rice. Our results indicated that ultrasound treatment did not affect free amino acid content in the late stages of germination, which may be related to the concentration of soluble protein in maize at this stage.

During germination, a large number of biological enzymes are activated and released, causing seeds to undergo a series of complex chemical and physiological changes. Proteins are hydrolyzed into small peptides and amino acids by the action of endogenous enzymes, and the products of these hydrolysis reactions are involved in either catabolism or anabolism to produce new proteins. Ultrasonic induction accelerates this process by changing the activity of endogenous enzymes, and ultrasonic waves can destroy the binding structures of protein and starch, change the spatial structures of proteins, increase the concentrations of protein hydrolysis substrates, and accelerate the generation of free amino acids in maize during germination.

### 3.3. Effect of Ultrasonic Induction Treatment on γ–Aminobutyric Enrichment in Germinating Maize

#### 3.3.1. Effect of Ultrasonic Induction Treatment on the Glutamic Acid Content of Germinating Maize

**Figure 5:**
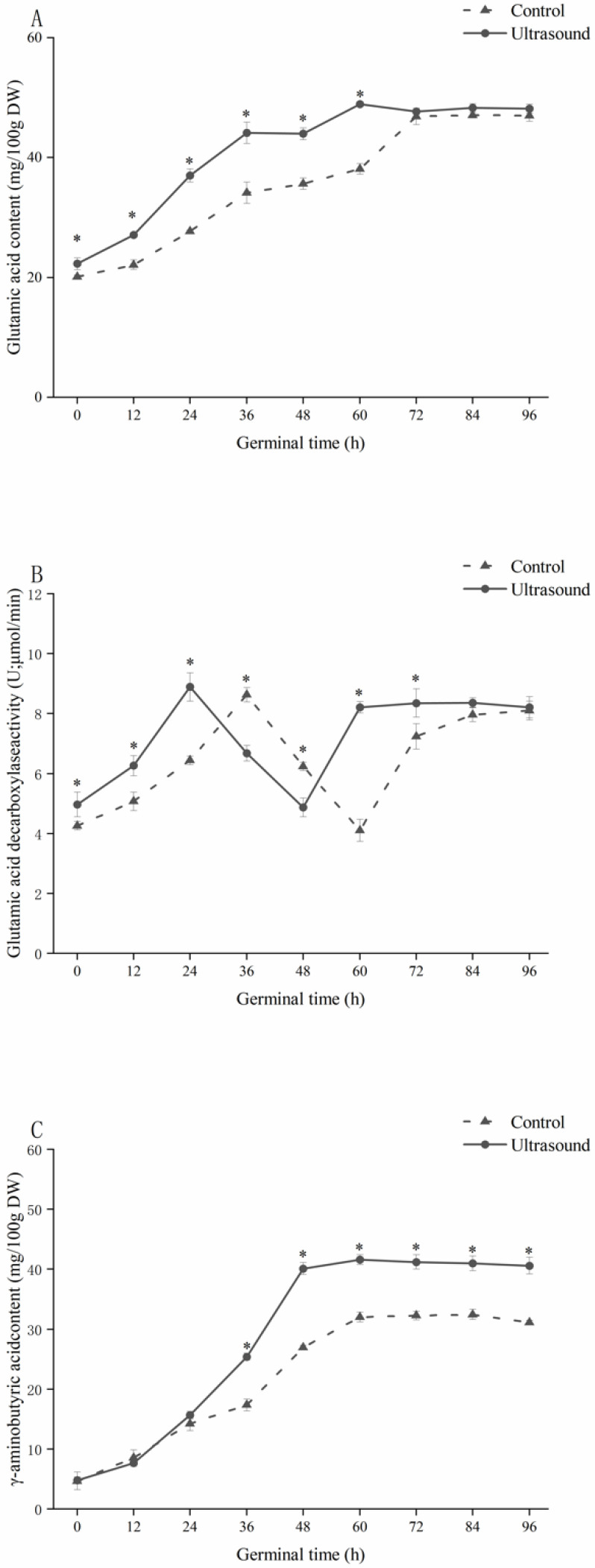
The effect of ultrasonic induction treatment on glutamic acid content (**A**), glutamic acid decarboxylase activity (**B**), and *γ*–aminobutyric acid content (**C**) during maize germination. * Indicates a significant difference (*p* < 0.05) between the ultrasound and control groups.

As shown in Figure 5A, the Glu content in maize continued to increase as germination time increased. The Glu content in the ultrasound group was 10.58–29.26% higher than the control group from 0 to 60 h. The Glu content in the ultrasound group reached a maximum value of 48.91 ± 0.25 mg/100 g at 60 h of maize germination. In the present study, maize Glu content continued to increase during maize germination, while Xu et al. [46] found that Glu content continued to decrease with germination time during the pre-germination period of naked oats, and Ma et al. [47] found that soybean Glu content decreased significantly after germination. Other studies have suggested that the changes in protein and amino acid contents follow species-dependent patterns [48]. The increase in Glu content in both the control and ultrasound groups slowed down in the later stages of germination due to the increased respiratory intensity and the increased dry matter consumption that caused increased Glu consumption. Similar results were found by Kamjijam et al. [49], who examined the Glu content of white and colored rough rice during germination.

Glu acts as an important substrate for intracellular amino acid metabolism, is directly involved in the assimilation and isomerization of ammonia, and is consumed during the synthesis of other amino acids [22,39]. Free Glu is mainly derived from three pathways: proteolysis, the glutamine synthetase cycle, and GABA transaminase reactions. The conversion pathway of Glu is mainly utilized to generate precursors for GABA synthesis [50]. The change in Glu content in the ultrasound group indicates that ultrasonic induction treatment can accelerate the rate of Glu synthesis and the conversion rate of Glu.

#### 3.3.2. Effect of Ultrasonic Induction Treatment on the Activity of Glutamate Decarboxylase in Germinating Maize

As shown in Figure 5B, in both groups, GAD activity initially increased, then decreased, and eventually began increasing again during germination, resulting in two peaks of GAD activity. The first peak of GAD activity was 8.63 ± 0.24 U at 36 h after germination in the control group, while the first peak of GAD activity was 8.89 ± 0.47 U at 24 h after germination in the ultrasound group. The changes in GAD activity in the ultrasound group occurred earlier compared to those in the control group, indicating that ultrasonic induction treatment could promote the GAD activation process.

The peak of GAD activity in the two groups in this study occurred when the Glu content increased most rapidly, and the Glu content increased at a slower rate during the same time period when GAD activity decreased. GAD activity fluctuates in a wide range during grain germination [45], and GAD activity is regulated by the concentration of Glu [22]. GAD is a Ca^2+^/calmodulin-dependent enzyme with a calmodulin-binding region [51], and ultrasonic induction treatment promotes the degradation of substances around the plant cell wall, increasing intracellular Ca^2+^ concentration and thus GAD activity [45].

#### 3.3.3. Effect of Ultrasonic Induction Treatment on γ–Aminobutyric Content in Germinating Maize

As shown in Figure 5C, the GABA content in both groups continued to increase as germination progressed, reaching a peak of 32.44 ± 0.87 mg/100 g in the control group at 84 h of germination and 41.58 ± 0.81 mg/100 g in the ultrasound group at 60 h. Towards the end of germination, the GABA content of maize in the ultrasound group was 40.60 ± 1.39 mg/100 g, which was 8.42 times higher than the GABA content of ungerminated maize and 30.55% higher than the GABA content of maize in the control group.

GABA is an important intermediate product of the tricarboxylic acid cycle (TCA cycle) branch, which is obtained from GAD catalyzed decarboxylation of Glu in plants. GABA is converted to succinic semialdehyde by transamination and then further converted to succinic acid, thus entering the TCA cycle [52]. The rapid increase in GABA in the early stages of germination in both groups was likely due to the increase in Glu concentration as well as the increase in GAD activity. As germination time progressed, the rate of GABA proliferation began to slow down after the peak of GABA content in both groups, which slowed the increase in Glu and decreased GAD activity at this stage, directly limiting the increase in GABA content (Figure 5A,B).

From 0 to 12 h of germination, the GABA content of maize in the ultrasound group was lower than that of the control group, likely due to the ultrasonic induction treatment accelerating GABA leaching [53]. From 36 to 96 h of germination, the GABA content of maize in the ultrasound group was significantly higher than that in the control group, which may be attributed to the promotion of protein hydrolysis and the activation of endogenous enzymes by ultrasound. Endogenous enzymes are thought to be activated relatively slowly during seed germination [54], and ultrasound can accelerate this process through cavitation. As well as thermal and mechanical effects, ultrasonic induction enhances GAD activity in maize. At the same time, the activation of protease and the destruction of protein molecules induced by ultrasonic waves accelerate the decomposition of protein in maize, so that the concentration of Glu, the precursor of GABA, is maintained at a high concentration; therefore, ultrasonic induction promotes the reaction of GABA generation by increasing the enzyme activity and increasing the substrate concentration, resulting in GABA proliferation in maize. Ultrasound has been shown to significantly increase the GABA content in plants by causing damage to cell interiors and lowering cytoplasmic pH, thereby favoring Glu decarboxylation over GABA transamination [54]; therefore, ultrasonic induction can reduce or slow down GABA metabolism in maize during germination.

Liu et al. [55] found that the GABA content of brown rice treated with ultrasound at 40 KHz power for 30 min was 39.76% higher after germination compared with that of germinated brown rice without ultrasound treatment. GABA content accumulation was also found to be increased more by low-frequency compared to high-frequency ultrasound. As the ultrasound frequency increases, localized high temperature zones may be generated, leading to partial degradation of GABA and a decrease in GABA content [56].

## 4. Conclusions

This study showed that ultrasonic induction treatment significantly increased germination rate, sprout length, and respiration in maize. These changes may be related to the accelerated metabolism of maize by ultrasonic induction treatment, resulting in accelerated consumption of dry matter and starch and significant increases in soluble proteins, free amino acids, Glu, GABA content, and GAD activity. The GABA content and GAD activity of maize treated with ultrasound were higher compared to the control group throughout the germination process, indicating that ultrasonic induction treatment could promote the germination of maize, accelerate the process of germination, and shorten the time of GABA enrichment in germinating maize. The results of this study can be applied to increase GABA content in germinating maize.

## Figures and Tables

**Table 1:** Factors and levels of the orthogonal experiment.

Levels	Factors		
	Ultrasound Frequencies/KHz	Ultrasound Time/min	Ultrasonic Temperature/°C
1	28	10	30
2	45	20	35
3	100	30	40

**Table 2:** Orthogonal experimental table L_9_3^3^ and the experimental results for each index.

Factors Number	Factors and Levels			Germination Rate/%
	Ultrasound Frequencies/KHz	Ultrasound Time/min	Ultrasonic Temperature/°C	
1	28	10	30	85.50 ± 3.79 b
2	28	20	35	83.50 ± 2.25 cd
3	28	30	40	84.50 ± 1.91 c
4	45	10	35	86.00 ± 2.58 b
5	45	20	40	86.50 ± 1.91 b
6	45	30	30	90.00 ± 1.63 a
7	100	10	40	83.00 ± 2.58 d
8	100	20	30	83.50 ± 3.00 cd
9	100	30	35	82.50 ± 1.00 d

Different letters indicate significant differences at *p* < 0.05.
